# The Neurological Significance of Abnormal Natural Killer Cell Activity in Chronic Toxigenic Mold Exposures

**DOI:** 10.1100/tsw.2003.98

**Published:** 2003-11-13

**Authors:** Ebere Anyanwu, Andrew W. Campbell, Joseph Jones, John E. Ehiri, Akpan I. Akpan

**Affiliations:** Neurosciences Research, Cahers Inc., Conroe, TX, USA

**Keywords:** toxigenic mold, mycotoxins, neuroimmune disorders, NK cells, public health, United States

## Abstract

Toxigenic mold activities produce metabolites that are either broad-spectrum antibiotics or mycotoxins that are cytotoxic. Indoor environmental exposure to these toxigenic molds leads to adverse health conditions with the main outcome measure of frequent neuroimmunologic and behavioral consequences. One of the immune system disorders found in patients presenting with toxigenic mold exposure is an abnormal natural killer cell activity. This paper presents an overview of the neurological significance of abnormal natural killer cell (NKC) activity in chronic toxigenic mold exposure. A comprehensive review of the literature was carried out to evaluate and assess the conditions under which the immune system could be dysfunctionally interfered with leading to abnormal NKC activity and the involvement of mycotoxins in these processes. The functions, mechanism, the factors that influence NKC activities, and the roles of mycotoxins in NKCs were cited wherever necessary. The major presentations are headache, general debilitating pains, nose bleeding, fevers with body temperatures up to 40° C (104°F), cough, memory loss, depression, mood swings, sleep disturbances, anxiety, chronic fatigue, vertigo/dizziness, and in some cases, seizures. Although sleep is commonly considered a restorative process that is important for the proper functioning of the immune system, it could be disturbed by mycotoxins. Most likely, mycotoxins exert some rigorous effects on the circadian rhythmic processes resulting in sleep deprivation to which an acute and transient increase in NKC activity is observed. Depression, psychological stress, tissue injuries, malignancies, carcinogenesis, chronic fatigue syndrome, and experimental allergic encephalomyelitis could be induced at very low physiological concentrations by mycotoxin-induced NKC activity. In the light of this review, it is concluded that chronic exposures to toxigenic mold could lead to abnormal NKC activity with a wide range of neurological consequences, some of which were headache, general debilitating pains, fever, cough, memory loss, depression, mood swings, sleep disturbances, anxiety, chronic fatigue, and seizures.

## INTRODUCTION

Patients with chronic exposures to environmental toxigenic molds manifest adverse health reactions, some of which are allergic in nature, while others are pathologically systemic. Although these health conditions are probably inter-related, the manifestation of immune mechanism disorders is one of the underlying factors to which the neurological significance of the effects is the object of the discourse. The metabolism of toxigenic molds is known to produce two major metabolites: one that is beneficial (antibiotic) to humans and animals, while the other is nonbeneficial (mycotoxins). Mycotoxins have been shown to cause several immunotoxicologically undesired effects on humans, especially in the anatomical and chemical alterations of cell functions.

Hence, in various published studies, it is reported that patients presenting with toxigenic mold exposures show immune system dysfunctions to which abnormal natural killer cells (NKCs) are consistently observed. NKCs are a group of peripheral lymphocytes that contain a population of large granular lymphocytes. They are called “natural killer cells” because of their propensity to exert cytotoxic effects on various antigenic targets. They do not express T- or B-cell markers; however, they consistently express fragment crystalline (Fc) receptors on the cell surface[1]. The association of mycotoxins with various adverse health effects cannot be defined by the tests of abnormalities in the immune system alone. This is because of the possibility of underlying medical history and individual constitution. Furthermore, the reproducibility of immunologic test abnormalities reported under these conditions has not been verified with any degree of confidence in terms of scientific methodology. It is very important that one understands the biologic variability and diurnal variations in lymphoid cell numbers in interpreting cellular immunologic profiles to achieve diagnostic accuracy.

The aims of this paper were to review and put together the pieces of evidence scattered in the literature that might help to fill some of the information gap, and to evaluate the neurological significance of abnormal NKC activity in patients presenting with chronic toxigenic mold exposure.

## METHODS

A comprehensive review of the literature was carried out to evaluate and assess the conditions under which the immune system could be dysfunctionally disrupted by chronic exposures to toxigenic molds. The functions, mechanism, the factors that influence NKC activities, and the roles of mycotoxins in NKC abnormalities was cited with pieces of evidence from the literature wherever necessary. This background led to the overall assessment, evaluation, and discussion of how chronic toxigenic mold exposures could influence the activity of NKCs.

## RESULTS OF REVIEW

The major presentations common to patients with chronic toxigenic mold exposures were headache, general debilitating pains, nose bleeding, fevers with body temperatures up to 40°C (104°F), cough, memory loss, depression, mood swings, sleep disturbances, anxiety, chronic fatigue, vertigo/dizziness, and in some cases, seizures. Although sleep is commonly considered a restorative process that is important for the proper functioning of the immune system, its deprivation was commonly reported in the literature. It is most likely that mycotoxins exert some rigorous effects on the circadian rhythmic processes resulting in sleep deprivation to which an acute and transient increase in NKC activity is observed.

Several data on the effects of mycotoxins including subjective and objective report assessments implicated sleep in the modulation of natural immunity and correlated with a reduction of NKC activity in major depression. Psychological stress had an influence on NKC activity, which played a central role in the protection against microbial infection and cancer. Anxiety was a typical type of psychological stress observed in patients with toxigenic mold exposures and was probably associated with mycotoxic activities. Transitory changes in NKCs associated with pain and stress were implicated in the development of infectious disease episodes after an acute stressful event. Although some studies suggested that acute severe pain without tissue injury markedly increased NKC cytotoxicity, there were some indications that patients presenting with chronic toxigenic mold exposures were likely to have tissue injuries due to mycotoxicity. It was also found that at very low physiological concentrations, mycotoxins significantly stimulated *in vitro* the antibody-dependent cellular cytotoxicity and NKC activities.

## DISCUSSION

### Functions of NKC

NKC activities are modulated by lymphokines and their proliferative and cytolytic activities are stimulated by interleukin-2 (IL-2) and enhanced by interferon (IFN)[1,2,3]. Combined depletions of NKCs and CD4(+) completely eliminated the antitumor effects. Also, NKCs play an important role in the activation of the potent immunotherapeutic effects elicited by intratumoral injections of IL-12 secreting dendritic cells (DCs) that are dependent on IL-12[4]. Although NKCs and DCs are two distinct cell types of innate immunity, *in vitro* interaction of human NKCs with autologous DCs results in DC lysis and the contact-dependent interactions between activated human NKCs and immature DCs (iDCs) provide a “control switch” for the immune system[5].

Hence, at low NKC/DC ratios, this interaction dramatically amplifies DC responses, whereas at high ratios it completely turns off their responses. Specifically, culture of activated human NKCs with iDCs, at low NK/DC ratios (1:5), led to exponential increases in DC cytokine production, which were completely dependent on cell-to-cell contact[5]. DC maturation was also driven by cognate interactions with NKCs and maturation was dependent on endogenously produced TNF-alpha in the culture. At slightly higher NKC/DC ratios (5:1), inhibition of DC function was the dominant feature due to potent killing by the autologous NKCs[5]. Impaired activation of NKCs and lymphokine-activated KCs has a role in the development of hepatocellular carcinoma[6,7,8].

### Mechanism of NKC Activity

One of the major mechanisms by which the immune response deals with foreign or abnormal cells is to damage or destroy them. Such immunologic cytotoxicity may lead to complete loss of viability of the target cells (cytolysis) or an inhibition of the ability of the cells to continue growing (cytostasis). Immunologic cytotoxicity is a principal mechanism by which the immune response copes with and often eliminates foreign materials or abnormal cells. Although it is accepted that the understanding of the mechanisms of NKC functions might lead to novel therapeutic strategies for the treatment of human disease, to date the mechanisms of human NKC activity have been poorly defined[9].

The fact that serotonergic systems can modulate cholinergic functions implies that both the cholinergic and serotonergic systems are necessary for eliciting the conditioned enhancement of NKC activity[10]. NKCs should no longer be thought of as direct cytotoxic killers alone as they clearly serve a critical role in cytokine production, which may be important to control cancer, infection, and fetal implantation. NKCs play a critical role in immune surveillance and cancer therapy. However, new insights into NKC biology have suggested major roles for NKCs in infection controls and uterine function[11,12].

## Effects of Mycotoxins on NKC Function

Mycotoxins significantly stimulate the proliferative response of splenocytes, thymocytes, T-cells mitogen Concanavalin A (Con A), and suppress the mitogenic responses to both lipopolysaccharide and Con A in prenatally exposed animals[13]. This indicated that prenatal exposure to relatively low-dose mycotoxins might induce immunosuppression[12,13,14,15,16]. The immunologic effects of Fusarium mycotoxins (deoxynivalenol [DON], 3-acetyldeoxynivalenol, fusarenon-X, T-2 toxin, zearalenone, alpha-zearalenol, beta-zearalenol, and nivalenol) on human peripheral blood mononuclear cells from different blood donors showed that T-2 toxin, fusarenon X, nivalenol, and deoxynivalenol exerted the highest immunosuppressing effect *in vitro*[17,18]. Mycotoxin-induced immunosuppression is manifested as depressed T- or B-lymphocyte activity. By the virtue of mycotoxin inhibition of NKC activity, the protection against tumor development may also be affected[18].

A study[19] of possible immunomodulative effects of aflatoxin B1 (AFB1) at concentrations lower than 5.10(-11) M or 0.05 ppb and 10(-5) M, respectively, on NKCs from healthy volunteers showed that AFB1 has an immunosuppressive effect on NKC activity. Various mycotoxins, such as gliotoxin, have immunomodulating activity. They selectively affect certain lymphocyte subsets, possibly through the mechanism of apoptosis, which is increased *in vivo* as well as *in vitro*[20]. Contrary to inhibitory effects, other mycotoxins (such as the Patulin mycotoxin produced by many fungal species of the genera *Penicillium*, *Aspergillus*, and *Bryssochamys*) were shown to increase the NKC and monocyte counts by 30 and 24%, respectively[21].

## Factors that Influence NKC Activities

NKC activity is influenced by a variety of factors including stress, chemical exposure, infections, chronic fatigue syndrome, immune deficiencies, and cancer. In an increasing number of patients with various diseases, IL-2, IFN, and NKC cytoxic factors have been shown to enhance NKC activity. Therefore, enhancement of IL-2 production may be useful in reactivation of NKCs in patients with chronic toxigenic mold exposures. Therefore, alterations of NKC functions by mycotoxins could be involved in the neuroimmune mechanism of neurological deficits found among the patients. In addition, alterations of NKC cytotoxicity control and NKC-derived cytokine release in mycotoxicity could be involved in the neuroinflammatory mechanism related to the progression of neurodegeneration in mycotoxicity. NKC activity has been associated with altered immunity and reduced occurrence of autoimmune diseases and malignancies.

It appears that an abnormal immunoregulatory response based on environmental mycotoxic damage to T cells is fundamental to the production of symptoms of mycotic illnesses[22]. That means that the stimulation of T cells or T-cell phenotypic subsets by environmental mycotoxins results in release of cytokines that can effect appropriate target cells of multiple organ systems, resulting in a wide range of neurological and behavioral symptoms[23]. It has been previously reported[24] that physiologic quantities of platelet-derived growth factor (PDGF) on their surface significantly inhibit human NKC cytotoxicity, and that patients with myelofibrosis and essential thrombocythemia have significantly elevated circulating levels of plasma PDGF. Pretreatment of normal NKCs with concentrated PDGF-containing, platelet-poor plasma from patients with these diseases significantly inhibits effects on the correlation between abnormal platelet release, plasma accumulation of PDGF, and the observed NKC immunodeficiency in these myeloproliferative patients if exposed to toxigenic molds.

## Neurological Significance and Sleep Disturbances

Based on the literature reviews, the major presentations in patients with chronic toxigenic mold exposures were headache, general debilitating pains, nose bleeding, fevers, cough, memory loss, depression, mood swings, sleep disturbances, anxiety, chronic fatigue, vertigo/dizziness, and in some cases, seizures. The effects of sleep deprivation on immune profile in human have been reported in the literature[25,26,27]. However, since it is accepted that sleep deprivation modifies different host defense activities and increases the susceptibility to various diseases, it is most likely that mycotoxins exert some marked effects on the circadian rhythmic process resulting in sleep deprivation to which an acute and transient increase in NKC activity is observed. In addition to immune dysfunction, sleep disturbances due to mycotoxins may increase hypothalamic-pituitary-adrenocortical (HPA) axis activity in depression[26]. Since sleep is restoratively important for the proper functioning of the immune system, it could be disturbed by mycotoxic activity.

Moreover, in view of basic evidence of a reciprocal interaction between sleep and cytokines and the correlation with declines in NKC cytotoxicity activity[28], the mycotoxic effects may increase the severity of depressive symptoms or the presence of a mood disorder[29]. This suggestion is supported by several data measured by either subjective report or electroencephalographic (EEG) assessment that correlated the reduction of NKC activity with major depression[28,29,30].

## Psychological Stress

Mycotoxicity can lead to psychological stress, and psychological stress has an influence on NKC activity, which plays a central role in protection against microbial infections and cancer. Anxiety is a typical psychological stress reported in a majority of chronic toxigenic mold exposures and anxiety and is associated with mycotoxic activities[31].

Transitory changes in NKCs associated with pain and stress were implicated in the development of infectious disease episodes after an acute stressful event[32]. Although acute severe pain without tissue injury markedly increased NKC cytotoxicity[33], indications in the review seemed to imply that patients presenting with chronic toxigenic mold exposures were more likely to have tissue injuries due to mycotoxicity. Hence, it was reported that mycotoxin in physiological concentrations ranging from 10(-11) M to 10(-9) M significantly stimulated *in vitro* the antibody-dependent cellular cytotoxicity and NKC activities in mouse leukocytes from axillary nodes, spleen, and thymus. It was also reported that mycotoxins induced change in IL-2 production, a rapid, transient and significant decrease of intracellular cyclic AMP at 30 s, inositol 1,4,5-trisphosphate levels at 30 and 60 s, and a stimulation of protein kinase C activity in membrane fractions after 5 min incubation, thus suggesting that inositol-phospholipid signaling and cAMP messenger systems are involved in the increase of mycotoxin-related NKC activity when leukocytes were incubated in the presence of mycotoxins (FB1)[33].

## Rules of Mycotoxins/NKC in Carcinogenesis

The combination of mycotoxins, e.g., fumonisin (FB1) and aflatoxin (AFB1), showed synergistic toxicological and carcinogenic effects in a short-term carcinogenesis model in the liver of experimental animals. In this example, both the AFB1 and FB1 compounds exhibited slow cancer initiating potency individually, however, in combination, the number and size of GSTP(+) lesions significantly increased as compared to the separate treatments. This combination could pose an increased risk in humans[34]. AFB1 is one of the most potent rodent and human liver carcinogens and on cytochrome P450–specific metabolism, it induces mutation as well as mitotic recombination events *in vitro* systems hence, supporting the hypothesis that mitotic recombination represents a central mechanism of action in AFB1-induced liver carcinogenesis[35,36,37]. The NKC activity may facilitate the development of malignant tumors such as lymphoma cells[38]. A monoclonal antibody recognizing a NKC-associated antigen was used to sequentially quantify numbers of peripheral blood NKCs in a small group of patients with large cell lymphoma[39]. A role for NKCs in the surveillance and control of abnormal lymphoproliferation was suggested, as was the possible prognostic utility of sequential peripheral blood NKC quantification in patients with large cell lymphoma[39,40].

## The Mechanism of Mycotoxic Carcinogenesis

The ubiquitous mycotoxin ochratoxin A (OTA), for example, is associated with the development of urothelial tumors and nephropathies in laboratory animals and in humans with stark species and sex differences with respect to susceptibility in disease development. The mechanism of action is probably by OTA-mediated disruptions in normal cell-cycle control and could be a major constituent of the mechanisms underlying both its carcinogenic and nephropathy-inducing activities[43]. Mycotoxins, especially OTA, specifically inhibit NKC activity and increase the growth of transplantable tumor cells without altering T-cell– or macrophage-mediated antitumor activity. Thus, ochratoxin appears to suppress NKC activity by inhibiting productions of basal interferon, thus suggesting a possible role for altered NKC function in the development of mycotoxin-induced carcinogenesis[14].

FB1 is epidemiologically linked to the high incidence of human esophageal cancer in South Africa and China and the mechanism is by inhibition of ceramide synthase and disruption of membrane phospholipids. The mechanism is also by the activation of protein kinase B which leads to increased survival, inhibition of GSK-3beta activity, and post-translational stabilization of cyclin D1, all events responsible for disruption of the cell cycle G (1)/S restriction point in hepatocytes[44,45]. Multiple signal transduction pathways such as PKC have been shown to play an important role in carcinogenesis. FB1 induces protein kinase C translocation via direct interaction with diacylglycerol site that also binds phorbol esters. Because phorbol esters are well-known tumor promoters, it is a more plausible cellular mechanism to explain the carcinogenicity of FB1[46,47]. Other studies suggested that the concept that FB1-induced mitogenesis might be the mechanism of carcinogenesis and that the primary target organs are the kidney and liver[48]. FB1 is also hepatocarcinogenic in rats, but epidemiological with esophageal cancer in humans[48,49].

## Mycotoxins, NKCs, and Chronic Fatigue Syndrome

Several reports showed that chronic fatigue was one of the characteristic manifestations of chronic toxigenic mold exposures. Chronic fatigue syndrome (CFS) is characterized by unexplained, debilitating fatigue or easy fatigability lasting longer than 6 months. While a viral basis of infection is proposed to be the cause of CFS, other viral infections do not generally persist after several weeks. It is now acknowledged that mycotoxicity leads to immunologic disorders including abnormal functions and distributions of T lymphocytes, B lymphocytes, NKCs, and monocyte/macrophages, and these disorders have been described in CFS. NKCs are known to play an important role in host resistance against fungal and viral infection as well as in the regulation of the immune systems[50].

## NKCs in Malignancies

NKCs are the subjects of great current interest because of their possible (*in vivo*) role in tumor cell surveillance and killing, and because of the potential application of cytokine-modulated NKCs in cancer immunotherapy. In addition, clonal proliferations of NKC-associated populations represent a high proportion of chronic (non-B) lymphoid malignancies and abnormal (both clonal and nonclonal) NKC-associated components are being increasingly reported in association with diverse clinical pictures such as autoimmune disease. This communication extensively reviews what is presently known regarding normal and leukemic NKC-associated phenotypic diversity, the mechanisms of NKC-mediated cytolysis, the role of NKCs in malignancy, and the diagnostic and cellular aspects of malignant NKC-associated proliferations[51]. Abnormal large granular lymphocytes appeared in peripheral blood and increased up to 17,000/microliters that had abundant pale cytoplasm with rich large azurophilic granules and a large nucleus with a few nucleoli. These cells did not have the activity of antibody-dependent cellular cytotoxicity but had natural killer activity[52].

Immunohistochemically, biopsy specimens taken from the lesional skin demonstrated that the NKC phenotype plays a role in the development of the abnormal skin reactions to mosquito bites and other systemic manifestations, suggesting that NKC lymphocytosis may show severe hypersensitivity to mosquito bites as the most outstanding manifestation[53]. In patients with primary biliary cirrhosis (a chronic, life-threatening disorder that is believed to be immunologically mediated), NKC function is low and poorly responsive to high concentration of IFN suggesting a kinetic impairment of NKC function in patients with PBC[54].

## NKC Activity in Experimental Allergic Encephalomyelitis

A decrease in NKC activity was detected at the onset of clinically defined experimental allergic encephalomyelitis (EAE) disease in long-tailed macaques (*Macaca fascicularis*) by inoculation of autologous myelin basic protein (BP) in complete Freund’s adjuvant[55]. There was a statistically significant lower NKC activity than normal controls in atopic patients when related to a low IL-2 production. Both facts inversely correlated with the concentration of IgE in serum. We concluded that atopic patients’ vulnerability to fungal infections represent probably a defective NKC activity. Suppressor T-cell function is abnormal in these patients. Both defects could be due to a faculty immunoregulatory helper function.

## CONCLUSIONS

Considering the dangers involved in attempting to make clinical evaluations based on results of isolated *in vitro* assessment of quantity or function of immune system cellular and humoral components, the medical history, physical examination, and objective neurological evaluations should be used as confounding variables that could affect the increase in the validity of the outcomes. In the light of this review, it is concluded that chronic exposures to toxigenic mold could lead to abnormal NKC activity with a wide range of neurological consequences some of which are headache, general debilitating pains, body temperature up to 40°C (104°F), cough, memory loss, depression, mood swings, sleep disturbances, anxiety, chronic fatigue, and seizures.

## ACKNOWLEGEMENT

The authors are grateful to the mothers who participated in this study. Development of this paper was made possible through financial support from the Catherine Elliott Fund, Liverpool School of Tropical Medicine, Liverpool, England, United Kingdom.

## BIOSKETCHES

**Ebere C. Anyanwu**, **Ph.D.,** is a Clinical Neurophysiologist and the Director of Cahers Neurosciences Research Inc., Houston, TX. He is also an Adjunct Professor of Anatomy and Physiology at the North Harris and Montgomery Colleges in Houston and the Woodlands, TX.

**Andrew W. Campbell**, **M.D.,** is an Expert Medical Scientist in Immunotoxicology and the Medical Director and Chief Executive of the Center for Immune, Environmental, and Toxic Disorders, Houston, TX. E-mail: md@immunotoxicology.com.

**Joseph Jones, Ph.D.,** is the Dean of Graduate School and Associate Provost for Research, Texas Southern University, Houston. E-mail: jones_jx@tsu.edu.

**John Ehiri, Ph.D.,** is an Assistant Professor in the Department ofMaternal and Child Health, School of Public Health, University of Alabama at Birmingham, USA. E-mail: jehiri@uab.edu.
